# Reappraisal of ischemia-reperfusion injury in a short duration laparoscopic surgery, a pilot study

**DOI:** 10.1186/s12893-021-01339-4

**Published:** 2021-09-20

**Authors:** Amitai Bickel, Soliman Khatib, Eli Kakiashvilli, Eilam Palzur

**Affiliations:** 1 Department of Surgery A, Galilee Medical Center, P.O.Box 21, 22100 Nahariya, Israel; 2 Azrieli Faculty of Medicine, Bar-Ilan University, Safed, Israel; 3 Department of Biochemistry, MIGAL Galilee Research Institute, Kiryat Shmona, Israel; 4 Eliachar Research Laboratories, Galilee Medical Center, Nahariya, Israel

## Abstract

**Background:**

Serum biochemical changes during laparoscopic surgery and positive pressure pneumoperitoneum (PP) may reflect mild oxidative stress due to the ischemia-reperfusion (I/R) mechanism. However, there is still a controversy regarding the exact mechanism of PP in creating oxidative stress and whether the induction of PP causes I/R effects at all. To elucidate this debated issue, we studied, for the first time, the changes of I/R parameters in the serum, in a pilot study, during laparoscopic cholecystectomy using a reliable, independent exogenous oxidative biomarker, together with common intrinsic biomarkers of oxidative stress.

**Patients and methods:**

Our study included 20 patients scheduled for elective laparoscopic cholecystectomy. We evaluated the levels of the extrinsic and endogenous markers for oxidative stress during awareness, under anesthesia, the end of surgery (abdominal CO2 evacuation), and 2 h afterward.

**Results:**

After an initial increase in oxidative stress following anesthesia, we did not notice any further significant rise in the levels of the synthetic exogenous and the endogenous biomarkers at the end of the surgery and 2 h later on. However, a positive correlation was noted between the levels of both the intrinsic and extrinsic markers.

**Conclusions:**

In our study, the capability of the extrinsic biomarker to detect mild oxidative stress was not validated. Our study stresses the heterogeneous nature of the oxidative reactions and the diversity of the endogenous and exogenous biomarkers while detecting various biochemical patterns under mild oxidative stress, during the short period of laparoscopic surgery.

## Introduction

Oxidative stress is an imbalance between processes that increase free radicals generation and those that reduce it by various anti-oxidative mechanisms in favor of the formers. Overproduction of reactive oxygen and nitrogen species is associated with structural and functional derangement of biomolecules such as nucleic acids, proteins, and lipids [1–4]. Oxidative stress is involved in various pathophysiological disorders, including cardiovascular, neurological malignant, and inflammatory changes. The oxidative components accumulate physiological, metabolic pathways during diverse disease states [5–8].

Early diagnosis of oxidative stress may be of significant clinical importance as it might promote early treatment**,** thus reducing associated morbidity and mortality.

Consequently, the need for reliable endogenic biological markers to characterize evolving oxidative stress is warranted to predict the early development of treatable pathological states. However, the mechanism that involves oxidative stress in diverse stress stated as surgery, trauma, and infection, is not completely clear. Various methods and endogenous biomarkers have already been suggested, based on products of thiobarbituric acid, isoprostanes, and oxysterol for unsaturated fatty acids, keto-proteins, and nitro-tyrosine for oxidative protein products, etc. These were neither specific nor could identify early oxidative stress [5, 9, 10]**.** The use of fatty acids binding proteins (FABS) and procalcitonin were suggested, too, for early diagnosis of intestinal ischemia and as a marker of intestinal damage [11, 12].

During laparoscopic surgery and the creation of positive pressure pneumoperitoneum (PP), abdominal CO2 insufflation (usually up to 14 mmHg) can lead to reduced abdominal perfusion (liver, kidneys, intestine, stomach) through a well-known mechanism. Mild ischemia and reperfusion that follows abdominal CO2 evacuation should lead to mild ischemia-reperfusion changes [13–18]. Eventually, oxidative stress during laparoscopic surgery might carry deranged clinical significance, particularly during prolonged operations in medically vulnerable patients. However, there is still controversy regarding the influence of PP on I/R effects during laparoscopic surgery, partially due to the heterogeneity of patient data and the diversity of surgery and biomarkers detected [17–24].

As oxidative stress can involve various organic compounds, there is a need for a reliable marker to identify the oxidative products of fatty, protein, and nuclear acids all together. In addition to the common endogenous biomarkers, an exogenous independent marker can be used. Such a sensitive exogenous biomarker has already been synthesized, built up by organic sub-units combined by covalent bonds. This biomarker is composed of amino acid tyrosine that combines linoleic acid by amidic bond. Cholesterol is combined with the carboxyl group of tyrosine to produce ester (linoleic tyrosine cholesterol ester). Nucleic acid (such as deoxy guanine) can link the molecule as well. This exogenous marker provides representation to the main human organic components [25]. The linoleic acid represents the polyunsaturated fatty acids, tyrosine residue stands for the proteins, and the hydrophilic sub-unit **2-**deoxyguanosine represents the DNA molecule. Each part of the synthetic marker can be oxidized to form specific products, depending on the type of reactive oxygen and nitrogen species present. Previous studies have demonstrated an association between elevated levels of this marker and various states of tissue ischemia that accompany diabetic complications, cerebral diseases, and atherosclerotic cardiovascular pathophysiology [1].

Our pilot study aimed to use an exogenous synthetic biomarker, together with the common intrinsic biomarkers, to detect oxidative stress during laparoscopic surgery to elucidate the controversy that still exists and relevant regarding oxidative stress under PP**.** We suggest that upon the reliability of the exogenous biomarker, it could be used to detect diverse clinical pathophysiology, such as early detection of intestinal ischemia.

## Patients and methods

Ethical approval for this study was provided by the Ethical Committee Institutional Helsinki committee of Galilee Medical Center, Nahariya, Israel (Chairman Prof. J. Bornstien; Ethical Committee N# 21311) on 14 June 2011. The study was performed in accordance with the relevant guidelines. Written informed consent was obtained from all patients.

### Study population

Our study group included 20 patients who were admitted electively to the surgical ward for laparoscopic cholecystectomy due to symptomatic cholecystolithiasis. The mean age of our study group was 55.6 ± 17.4 years (range 31 to 77), and all were in good medical condition (ASA I-II). Mild ischemic heart disease was noted in two patients and controlled hypertension in six patients.

### Surgery

Surgery was done by the usual laparoscopic technique, under general anesthesia, while the patients are lying on a 15-degree reverse Trendelenburg position. The abdomen was insufflated by CO2 gas up to a pressure of 14 mmHg., under digital control. The mean time of PP was 40.4 ± 14.6 min (range 24 to 68 min.). After introducing four cannulas into the peritoneal cavity, the cystic duct and vessels were identified, clipped, and cut, followed by dissection, resection, and gallbladder extraction of the abdominal cavity, to conclude the operation.

### Blood collection and analysis

Before surgery and the morning of the first postoperative day, routine blood tests were done and included complete blood count, glucose, creatinine, urea, amylase lipase, electrolytes, and liver function tests. Blood analyses of the exogenous marker (1 cc) were done before surgery (awake state and at the end of anesthesia), at the end of laparoscopic surgery (abdominal CO2 evacuation), and about 2 h later.

### Methods to detect the markers

The technique using the exogenous marker has already been detailed in the scientific literature [25]. In brief, blood samples (1 ml) were collected in a glass tube containing heparin and 8 µl of the exogenic marker (from a stock solution of 20 mM marker dissolved in DSMO) or DSMO alone (control) and left to coagulate at room temperature [1, 26, 27]. After 1 h, blood was extracted twice with a 3ml mixture of organic solvents of hexane:2-propanol (3:2 v/v) each, containing 10 ppm (0.02%) butylated hydroxyl toluene. Following centrifugation**,** the organic phase was collected, and the solvent was evaporated under nitrogen until dryness. The samples were kept under argon at −20 ℃ until analysis. Before analysis, samples were re-suspended with 20% methanol in acetonitrile and divided into two parts for liquid chromatography-mass spectrometry (LC/MS) and gas chromatography-mass spectrometry (GC/MS) analyses. Initial separation and detection of compounds were performed by LC/MS analysis (for LT marker and its oxidized products) using high-pressure liquid chromatography (HPLC) and a Waters photodiode array detector. The compounds were eluted using a gradient of solutions A (0.1% acetic acid in acetonitrile) and solution B (0.1% acetic acid in double-distilled water). MS/MS analysis of the oxidized products was performed in a multiple-reaction monitoring scan mode using negative ions electron spray. Peak spectra were monitored between 30-600 m/z. A calibration curve of LT and LTG was run with each set of analyses [1, 27]. Gas chromatography (GC)/MS analysis for oxysterol detection was performed using high-pressure gas chromatography [1]. Dried extracts were subjected to the silyating reagent (BSA, 200 µl), and 1,4-dioxane (dried on 4 A molecular sieves and passed through 200 µ aluminum oxide) as a solvent, and heated to 80 ℃ for 60 min. Helium was used as the carrier gas. GC/MS detected samples in total monitoring mode [27]. For maximum sensitivity, the oxysterols were injected as their silyl ether derivatives, and the response factor for each oxysterol under the analytical conditions was calculated from the peak area ratio.

All the above analyses were done in MIGAL laboratories Center, Kiryat-Shmona, Israel.

In addition to our novel exogenous marker, various endogenous markers of oxidative stress were detected simultaneously as a reference. Those markers included: 7α-Hydroxylated cholesterol (7α-OH), 7β-Hydroxylated cholesterol (7β-OH), 5,6 α-epoxy cholesterol (α-epoxy), 5,6 β-epoxy cholesterol (β-epoxy), 7-ketocholesterol, 25-Hydroxylated cholesterol (25-OH), 27-Hydroxylated cholesterol (27-OH). The exogenous markers were labeled: OOH1 (representing cholesterol derivatives), Epoxy (representing the proteins LT), and 526 (stands for the nucleic acid).

### Statistical analysis

Statistical analysis was done using IBM SPSS statistics 19, and Quantitative variables were described by means, medians, range, and standard deviation. Frequencies and percentages detailed qualitative variables.

Regarding the changes in the levels of the exogenous and endogenous markers, we compared the data between every successive stage, as well as between the first and the last stage (first stage—wakefulness before surgery, followed by anesthesia, the end of laparoscopic surgery, and 2 h later). The Wilcoxon signed-rank test was used to characterize our findings. Comparison between different groups was carried out by one-way analysis of variance test (ANOVA). A p-value of 0.05 or less was considered to be of statistical significance. Graphs were plotted to delineate the results (line bar). Every subject's first measure served as his control. The various markers were expressed as their relative levels and the rate/percentage of change (and not regarding their absolute quantity) to enable a more straightforward comparison with the changes of the various phases during surgery.

## Results

As was detailed in the previous section, 20 patients were involved in the study. The mean age of our study group (20 patients) was 55.6 ±17.4 years (range 31 to 77), and all were in good medical condition (ASA I-II). Mild ischemic heart disease was noted in two patients and controlled hypertension in six patients. Surgical procedure and postoperative sequelae were without complications, and no hemodynamic instability was noted. Laboratory analysis, including complete blood count, liver and renal function tests, were all within normal limits before surgery and on the first postoperative day.

**Fig. 1:**
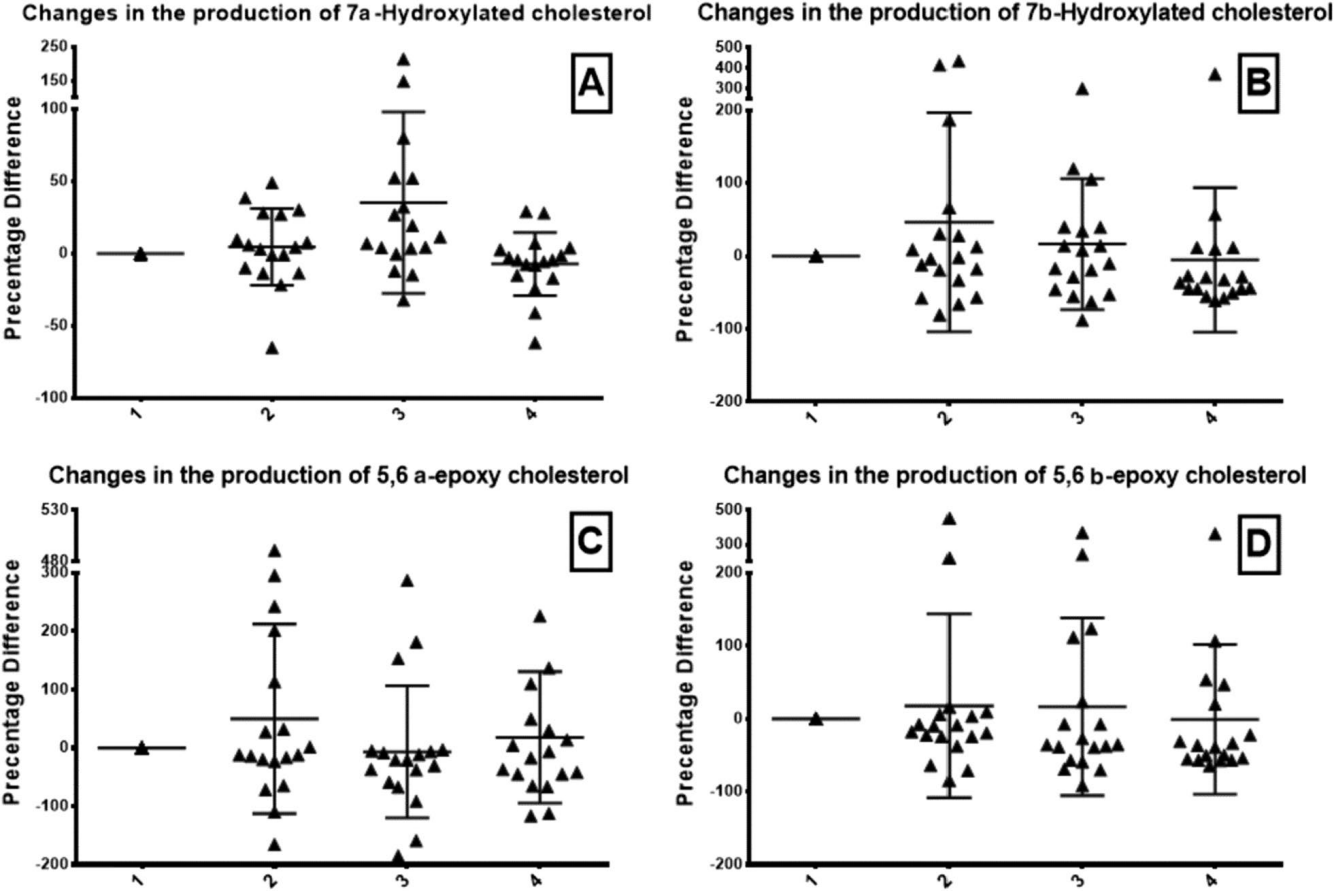
Endogenous markers: Changes in the levels of oxysterols products: 7a-Hydroxylated cholesterol (**A**), 7b-Hydroxylated cholesterol (**B**), 5,6 a-epoxy cholesterol (**C**), and 5,6 b-epoxy cholesterol (**D**). The four phases include the awake state (1), end of anesthesia (2), end of laparoscopic surgery (3), and 2 h postoperatively (4)

**Fig. 2:**
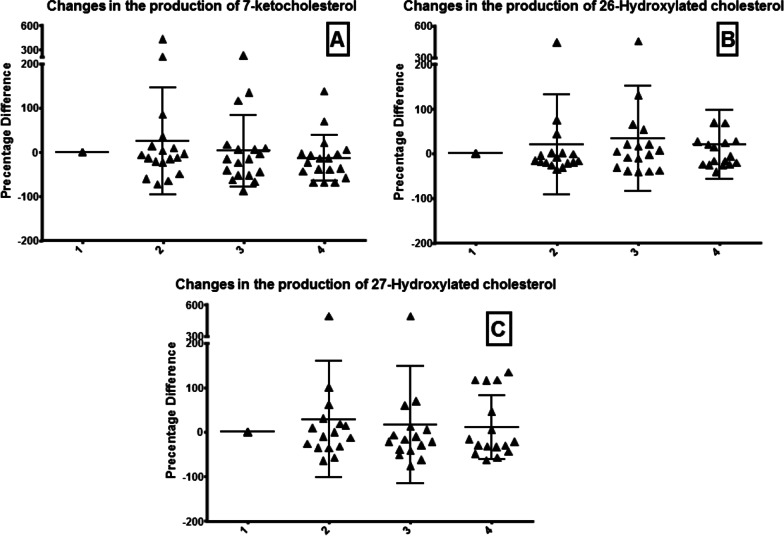
Endogenous markers (cont.): Changes in the levels of oxysterols products: 7-ketocholesterol (**A**), 26-Hydroxylated cholesterol (**B**), and 27-Hydroxylated cholesterol (**C**). The four phases include the awake state (1), end of anesthesia (2), end of laparoscopic surgery (3), and 2 h postoperatively (4)

**Fig. 3:**
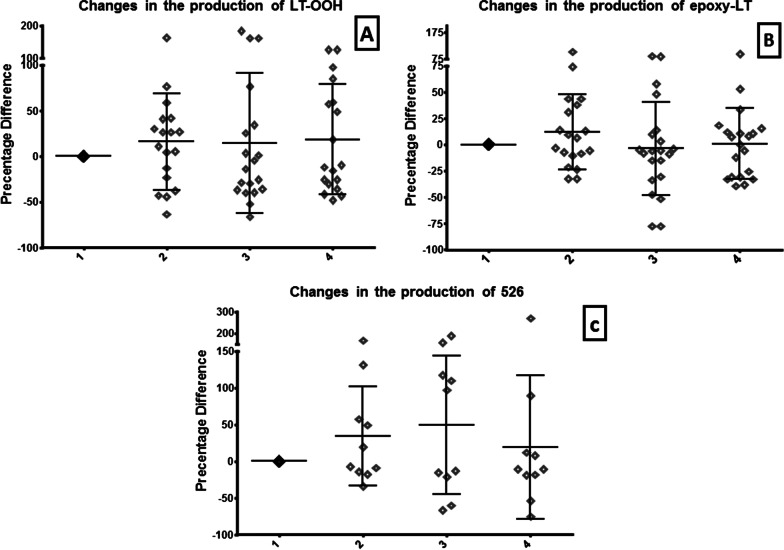
Changes in the levels of exogenous marker products: LT-OOH (**A**), epoxy-LT (**B**), and in the production of 526 (**C**). The four phases include the awake state (1), end of anesthesia (2), end of laparoscopic surgery (3), and 2 h postoperatively (4)

Our study did not detect a significant increase regarding the levels of the exogenous marker at the end of the surgery and 2 h afterward, following CO2 abdominal evacuation. However, we noted a mild increase in the oxidation marker levels during anesthesia, before surgery, and its descent afterward. The above results were in accordance with those of the endogenous oxidative products of the cholesterol derivatives molecules that were also detected in parallel (Table 1, Figs. 1, 2, 3). However, it should be expected that elevated reactive oxygen and nitrogen species (ROS/RNS) will be noticed during the postoperative period whenever oxidative stress exists.

**Table 1:** Levels of the exogenous and endogenous oxidation biomarkers during the four stages of surgery (laparoscopic cholecystectomy)

	Stage			
	1	2	3	4
Endogenous markers				
7α-Hydroxylated cholesterol (7α-OH)	0.0 ± 0.0	4.661 ± 26.64	35.27 ± 62.81	−7.150 ± 21.84
		ns	ns	Ns
7β-Hydroxylated cholesterol (7β-OH)	0.0 ± 0.0	46.47 ± 150.3	16.48 ± 89.68	−5.217 ± 99.05
		ns	ns	Ns
5,6 α-epoxy cholesterol (α-epoxy)	0.0 ± 0.0	49.95 ± 162.5	−6.604 ± 113.5	18.14 ± 112.8
		ns	P ≤ 0.0404	Ns
5,6 β-epoxy cholesterol (β-epoxy)	0.0 ± 0.0	17.72 ± 126.1	16.49 ± 121.8	−0.7928 ± 102.9
		ns	ns	P ≤ 0.0337
7-ketocholesterol	0.0 ± 0.0	25.27 ± 120.9	3.147 ± 80.84	−13.93 ± 51.65
		ns	ns	P ≤ 0.0482
25-Hydroxylated cholesterol (25-OH)	0.0 ± 0.0	20.69 ± 112.6	33.70 ± 118.0	20.39 ± 77.54
		ns	ns	Ns
27-Hydroxylated cholesterol (27-OH)	0.0 ± 0.0	28.48 ± 130.8	16.60 ± 132.3	10.37 ± 71.19
		ns	ns	Ns
Exogenous markers				
Oxidized N-linoleoyl tyrosine (hydroperoxide) (LT-OOH)	0.0 ± 0.0	16.09 ± 53.15	14.80 ± 76.74	18.64 ± 60.36
		ns	ns	Ns
Oxidized N-linoleoyl tyrosine (epoxide)(LT-epoxy)	0.0 ± 0.0	12.25 ± 35.88	−3.805 ± 44.61	0.9652 ± 33.81
		ns	ns	Ns
526	0.0 ± 0.0	34.22 ± 67.56	49.16 ± 94.02	19.20 ± 98.14
		ns	ns	Ns

## Discussion

Our study attempted to provide an additional perspective to elucidate the existing controversy regarding oxidative stress during laparoscopic surgery and PP due to the heterogeneous data concerning this relevant issue. For the first time, we used a sensitive, independent exogenous biomarker, in addition to the common endogenous biomarkers, to assess mild oxidative stress that frequently follows laparoscopic surgery and PP. The study assumption was that the induction of PP with increased intra-peritoneal pressure would lead to oxidative stress that should be detected by an objectively independent exogenous marker that had already been validated in various clinical disorders. The presence of mild oxidative stress during laparoscopic surgery has already been shown in previous studies and is the consequence of the influence of CO2 PP on the cardiovascular system. The creation of PP may lead to decreased visceral perfusion of the gastrointestinal tract along with the liver and kidneys, secondary to a reduction in cardiac venous return, increased systemic vascular resistance, and various associated endocrine and adrenergic changes [13–18, 28–30].

As was reported in the current study, we did not notice significant changes regarding the exogenous biomarker and the various endogenous markers that were detected following PP and surgery. However, we noticed a mild initial increase in the various markers immediately following anesthesia, probably due to its marked effects on the cardiovascular system, causing changes in perfusion.

It can be concluded that the exogenous marker, as well as the diverse endogenous markers, were slightly influenced by the marked cardiovascular changes that followed the pharmacological effects of anesthesia but were not sensitive enough to the mild ischemia-reperfusion changes following PP that accompanied the relatively short-term laparoscopic surgery. On the other hand, our results reflect the possibility that indeed no significant changes in oxidative stress exist during short-term laparoscopic surgery. Regarding the inability of the endogenous markers used in this study to detect mild I/R changes, several explanations are possible, including the use of markers that do not reflect entirely and correctly the biochemical reactions that follow PP. As is evident according to many studies, in humans as well as in animals, consensus (whether there is significant oxidative stress during laparoscopic surgery) has not yet been established due to diverse biomarkers heterogeneity, different study designs, and various methods of detection, which makes meta-analysis impossible [19–24, 31–36].

It can be concluded that as oxygenation by diverse free radicals can lead to heterogeneous oxygen byproducts, the techniques to detect oxidative stress in the serum should be expanded to reflect the broad oxidative spectrum. The absence of a significant increase in the exogenous and the endogenous markers in our study probably does not negate the presence of oxidative stress during PP. The endogenous markers in our study, for example, detected mainly sterols oxidation of cholesterol. In summary, although we did not notice significant changes in oxidative stress, it is relevant to short-term laparoscopic surgery, and in spite of the small-size design, it reached statistical significance.

We have deliberately chosen relatively short laparoscopic procedures that will reflect mild ischemia/reperfusion changes typical to early ischemic changes. Additionally, by using a novel exogenous biomarker for such a purpose in our study, it was better (to our opinion) to begin with the most common laparoscopic surgery (laparoscopic cholecystectomy), which is usually of a relatively short duration. Although our group of subjects is relatively small (20 patients), we did not feel it was necessary to expand it as our results were consistent, and have reached a statistical significance. However, further research is necessary and should involve detecting the exogenous marker during prolonged endoscopic surgery in addition to interventions that involve de-vascularization of intra-abdominal organs, and increased number of participants.

## Data Availability

The datasets used and/or analysed during the current study available from the corresponding author on reasonable request.
